# Authors' Reply: Concerns About the Generalizability Associated With a South African Randomized Controlled Trial on Prenatal Mothers

**DOI:** 10.2196/55930

**Published:** 2024-02-12

**Authors:** Maya Adam, Vān Kính Nguyễn

**Affiliations:** 1 Department of Pediatrics Stanford University School of Medicine Stanford, CA United States; 2 Institute of Global Health Heidelberg University Heidelberg Germany

**Keywords:** maternal child health, mHealth, mobile health, randomized controlled trial, short animated storytelling, South Africa, video health messaging

We are responding to a letter to the editor [1] regarding our recent article, “Effect of Short, Animated Video Storytelling on Maternal Knowledge and Satisfaction in the Perinatal Period in South Africa: Randomized Controlled Trial” [2].

Unfortunately, we believe the letter’s author (LA) may not have read the paper as carefully as they have claimed, and because of this, we find their criticisms to be somewhat misguided. Please see our detailed responses below.

The LA’s comment that the “study may not truly reflect the situation of patients in the real world” because we did not choose to use propensity score matching is unfounded. Propensity score matching has many applications [3]; one of them is matching to conduct *quasi*–randomized controlled trials. Large-scale and *ad hoc* population trials could benefit from a propensity score matching approach, but this was not the aim of our study. Importantly, one applies this type of matching to ensure that the background variables are similarly distributed among the comparison groups. In our study, we conducted proper random assignment of the participants and found that, quoting the letter, “no significant differences were seen between the SAS Intervention group and the control group” (Table 1). Thus, while we did not control for all the potential covariates, our design provides the highest level of confidence to identify the intervention effects. We do not see a link between the statistical test used in this study and the LA’s statement that the study “may not truly reflect the situation of patients.” We respectfully refer the LA to the Statistical Analysis section of our paper [2] where we detail why we used the beta-binomial model to describe the score distribution and control for covariate effects instead of a nonparametric univariate analysis of the knowledge score.

The LA’s comment advising us to use generalized estimating equations (GEEs) [4] has several unfounded statements: there are no longitudinal data so GEEs would not be advisable here, GEEs are a form of multivariate regression analysis, generalized linear model (GLM) was already used in our analysis, simply using GLM does not address whether a curvilinear relationship exists, and it is not necessary to use GLM to model curvilinear relationships.

Finally, while we agree that environmental factors might affect marital satisfaction, this is unrelated to our paper. The LA misread and did not realize that we measured *maternal* satisfaction with the video intervention; however, our main outcome was not maternal satisfaction but the knowledge score. The LA also erroneously states that “subgroup analyses were not conducted based on factors such as age, race, education level, and income. Hence, this paper would be more meaningful if these factors were analyzed in layers.” In Figure 3 of our paper [2], we show the results of our multivariable regression analysis, including the factors listed by the LA.

## Abbreviations

GEE: generalized estimating equation
GLM: generalized linear model
LA: letter’s author
